# Radiological manifestation of familial acute necrotizing encephalopathy with *RANBP2* mutation in a Far-East Asian family: Case report

**DOI:** 10.1097/MD.0000000000025171

**Published:** 2021-03-26

**Authors:** Yu-Jung Park, Jae-Yeon Hwang, Yong-Woo Kim, Yun-Jin Lee, Ara Ko

**Affiliations:** a Department of Radiology, Pusan National University Yangsan Hospital, College of Medicine, Pusan National University; b Research Institute for Convergence of Biomedical Science and Technology, Pusan National University Yangsan Hospital; c Department of Pediatrics, Pusan National University Children's Hospital, Pusan National University School of Medicine, Yangsan, Korea.

**Keywords:** child, encephalopathy, familial acute necrotizing encephalopathy, pediatric, radiology, *RANBP2*

## Abstract

**Rationale:**

Acute necrotizing encephalopathy (ANE) is a specific type of encephalopathy usually followed by febrile infection. It has an aggressive clinical course; however, it usually does not recur after recovery in cases of spontaneous ANE. Nevertheless, there are several studies reporting recurrences in familial ANE with RAN-binding protein 2 (*RANBP2*) mutation. There are few cases of familial ANE with *RANBP2* mutation in Asian populations.

**Patient's concerns:**

A 21-month-old Korean boy who was previously healthy, presented with seizure following parainfluenza – a virus and bocavirus infection, followed by 2 recurrent seizure episodes and encephalitis after febrile respiratory illnesses. Meanwhile, his 3-year-old sister had focal brain lesions on neuroimaging studies when evaluated for head trauma. The siblings also had an older brother who presented status epilepticus after febrile respiratory illness at the age of 10 months old.

**Diagnosis:**

Brain magnetic resonance imaging was performed to evaluate the seizure and neurologic symptoms. Imaging findings showed variable spectrum – from non-specific diffuse white matter injury pattern to typical “tricolor pattern” of the ANE on diffusion-weighted images. The other 2 siblings showed focal lesions in both external capsules and severe diffuse brain edema. Genetic tests identified a heterozygous missense mutation in the *RANBP2* [c.1754C>T (p.Thr585Met)] in 2 siblings and their mother.

**Interventions:**

Patients were treated conservatively with anticonvulsive agents, intravascular immunoglobulin, and steroids.

**Outcomes:**

Among the 3 siblings, 2 male siblings died from familial ANE, whereas the female sibling was asymptomatic.

**Lessons:**

These cases highlight the radiological aspects of familial ANE with incomplete penetrance of the *RANBP2* gene in 3 family members, showing variable involvements of the brain and natural history on magnetic resonance images. Radiologists should be aware of the typical and atypical imaging findings of familial ANE for prompt management of affected patients.

## Abstract 2

Supplemental Digital Content is available in the text

## 1 Introduction

Acute necrotizing encephalopathy (ANE) is a specific type of encephalopathy manifested with brain dysfunction usually followed by antecedent febrile infection. It has an aggressive clinical course with high mortality; however, it usually does not recur after recovery in cases of spontaneous ANE.^[1]^ Nevertheless, there are several studies reporting recurrences in familial ANE with RAN-binding protein 2 (*RANBP2*) mutation.^[2,3]^ To the best of our knowledge, there are few cases of familial ANE with *RANBP2* mutation in Asian populations.^[2,4]^ The authors recently experienced familial ANE episodes with a missense mutation in *RANBP2* [c.1754C > T: p.Thr585Met] presented with a wide spectrum of neuroimaging features; the imaging findings included extensive white matter injury patterns, multifocal brain lesions, and severe diffuse brain swelling.

Since the previous reports^[2,4]^ are clinically focused, the authors report cases of familial ANE in 3 members of a Far-East Asian family, focused on various radiological manifestations.

## 2 Case presentation

The Institutional Review Board of Pusan National University Yangsan Hospital approved this retrospective review of patients’ data and publication of this case study. The institutional review board waived the requirement for informed consent.

### 2.1 Case 1

A 21-month-old Korean boy who was previously healthy, presented with seizure and vomiting after 6 days of febrile respiratory illness. Patients had no previous history of neurologic disease, and developmental status was normal. Neurologic examinations revealed drowsy mental status, small pupil size, and sluggish pupil reflex. Laboratory findings were positive for parainfluenza – a virus and bocavirus on the real-time polymerase chain reaction (PCR). Other laboratory findings, including cerebrospinal fluid (CSF) analysis, were not specific.

**Figure 1:**
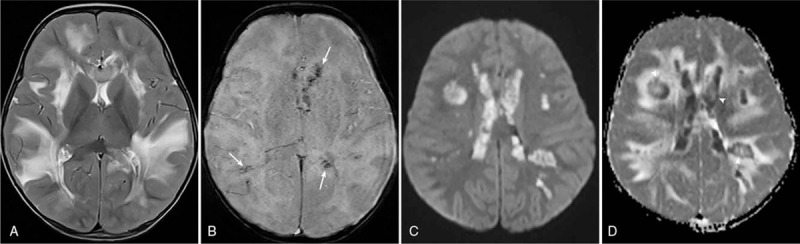
Initial neuroimaging findings of Case 1 presented with fever and seizure after parainfluenza – a virus and bocavirus infection. (a) Axial T2-weighted images demonstrate widely distributed T2 hyperintensity in both the cerebral hemispheres predominantly involving the white matters while sparing both the basal ganglia and thalami. (b) Petechial hemorrhages are demonstrated on SWIs (arrows). (c) On DWIs (*b* = 1000 s/mm^2^), lesions show multifocal diffusion restrictions in both cerebral white matters. (d) On the ADC map, the lesions show “tricolor pattern” or “target appearance” (arrow heads), which represent central necrosis and hemorrhage, cytotoxic edema of the middle portion, and outer vasogenic edema. ADC = apparent diffusion coefficient, DWI = diffusion-weighted image, SWI = susceptibility-weighted image.

Initial brain magnetic resonance imaging (MRI) showed T2 hyperintensity in both the cerebral hemispheres, involving the white matter and corpus callosum with multifocal areas of diffusion restriction with surrounding vasogenic edema. Petechial hemorrhages were detected on susceptibility-weighted images (SWIs). Lesions showed high signal intensity with high apparent diffusion coefficient (ADC) values at the center of the lesion, middle-low ADC value area, and outermost high ADC value area. Lesions did not show enhancement after gadolinium administration. Both the basal ganglia and thalami were spared (Fig. 1). Initial differential diagnoses included hypoxic injury, status epilepticus-related change, brain injury-related to viral infection, and metabolic disease.

After treatment with anticonvulsive agents, intravenous immunoglobulin (IVIG), and steroids, the patient achieved clinical improvement and was discharged with moderate motor weakness. The Bayle test performed 2 months later showed mild to moderate delay in the motor, social-personal, language, and cognitive domains. Follow-up MR images obtained 19 days after initial presentation showed resolution of diffusion restriction in both the cerebral hemispheres.

**Figure 2:**
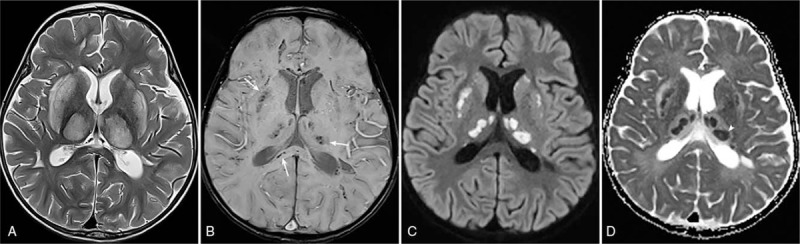
Recurrent episode of ANE in Case 1 following febrile respiratory illness. (a) Axial T2-weighted image shows symmetrical and bilateral T2 hyperintensity involving both the thalami and basal ganglia. The brainstem (images are not shown) was also involved. (b) SWI shows petechial hemorrhages (arrows). (c) and (d) DWI shows multifocal areas of diffusion restriction in both the basal ganglia and thalami; the ADC map shows the tricolor pattern (arrow heads). ADC = apparent diffusion coefficient, ANE = acute necrotizing encephalopathy, DWI = diffusion-weighted image, SWI = susceptibility-weighted image.

After 5 months, the patient visited our hospital again due to seizure and vomiting after 5 days of febrile respiratory illness. All family members were diagnosed with influenza – a virus infection in an outside hospital. Mental status was alert, but rigidity was noted on the neurologic examination. Brain MRI showed symmetrical and bilateral T2 hyperintensity involving the thalami, caudate nuclei, basal ganglia, and brain stem with diffusion restriction and petechial hemorrhage and similar ADC characteristics to the initial MR examination. The lesions showed faint enhancement after gadolinium administration (Fig. 2). Based on the characteristic neuroimaging findings, diagnosis of ANE was suggested.

Clinical improvement was achieved after treatment with IVIG and steroid. Denver test performed 7 months later showed a severe delay in the motor, social-personal, and language domains.

After 6 months of the second episode, patients had a recurrent ANE episode after influenza-a virus infection. Clinical manifestations and MRI findings were similar to those of the second episode, except for the involvement of the brain stem predominantly in the pons. Despite treatment with IVIG and steroid, he continued to deteriorate, and pupil reflex was lost. He expired on the third day of admission.

### 2.2 Case 2

At the same time of the second visit of Case 1, a 3-year-old girl visited our hospital with dysarthria, gait abnormality, and limitation of the right lateral gaze after head trauma. The patient was the older sister of Case 1. As noted earlier, she was positive for influenza – a virus on the real-time PCR.

Laboratory findings and CSF analysis were within the normal range. Although she had neurologic abnormalities including dysarthria, gait abnormality, and limitation of right lateral gaze at that time of trauma, these symptoms were regarded as trauma-related manifestations because subarachnoid hemorrhage in the left cerebral convexity was detected on head computed tomography (CT). The brain MRI showed symmetrical T2 hyperintensities involving the external capsules, right midbrain, and pontine tectum, and these lesions were accompanied by subtle petechial hemorrhage on SWI. The lesions showed ill-defined enhancement after gadolinium administration (Supplementary Fig. 1, http://links.lww.com/MD/F929).

The patient was discharged with conservative management. She did not present any ANE episode on follow-up even when she presented fever after influenza-a virus infection after 9 months.

### 2.3 Case 3

Familial history was retrospectively reviewed in detail in suspicion of familial ANE. The siblings had an older brother who presented with status epilepticus after febrile respiratory illness at the age of 10 months old, 5 years before the initial presentation of Case 1. All laboratory studies were nonspecific. Neurologic examination revealed comatose mentality, absence of spontaneous respiration, fixed pupil dilatation, the absence of light reflex, and flaccid muscle tone. Initial CT showed extensive brain edema. MRI performed after 2 weeks of hospitalization showed diffuse hyperintensities of the cerebral white matters with the absence of flow void of the intracranial vessels on spin-echo sequences (Supplementary Fig 2, http://links.lww.com/MD/F930). He fulfilled brain death criteria on the first day of admission and finally expired 2 months later.

### 2.4 Genetic study and outcomes

During the second seizure episode in case 1, genetic counseling proceeded in suspicion of familial ANE. Whole exome sequencing in case 1, case 2, and their parents, and the genetic testing revealed a heterozygous missense mutation in the *RANBP2* gene [c.1754C>T (p.Thr585Met)] in case 1, case 2, and their mother (Supplementary Fig 3, http://links.lww.com/MD/F931). Bringing the familial history, clinical settings, and radiological imaging findings altogether, the siblings were finally diagnosed with familial ANE. Among the familial members, case 2 and their mother had not experienced clinically apparent ANE, while the other 2 siblings expired due to severe ANE episodes.

## 3 Discussion

The authors reported various clinical and radiological manifestations of familial ANE in 3 siblings. Although most of the ANE cases are sporadic, Neilson et al reported autosomal dominant inheritance of ANE and identified the missense mutation in the *RANBP2* gene as a genetic susceptibility for ANE after febrile illness.^[5]^ In this case, 3 family members, 2 siblings, and their mother had *RANBP2* mutations. It is a limitation that we could not obtain the genetic test result of Case 3 child. Though the authors could not perform a genetic study for Case 3, the patient likely had familial ANE with *RANBP2* mutation based on the clinical manifestations, radiological findings, and autosomal dominant inheritance of familial ANE.

The clinical course of ANE can be categorized as the prodromal stage, acute encephalopathy, and recovery stage. Prodromal symptoms of ANE are non-specific and diverse due to viral infections, including fever, upper respiratory tract infection, chest infection, diarrhea, vomiting, and headache. With developing ANE, seizure, disturbance of consciousness, and focal neurologic deficits may present as brain dysfunctions.^[6]^ The clinical course of ANE is also diverse from a mild form of encephalopathy to a fulminant form leading to death.^[2,6–9]^

The inheritance of *RANBP2* mutation is autosomal dominant; however, incomplete penetrance has been reported with a 40% penetration rate.^[8,9]^ Disease onset, presentation, and manifestations can be diverse even among the family members with the same *RANBP2* gene mutations, even in a pair of identical twins.^[8,10]^ Several reasons have been postulated to explain the incomplete penetrance. Neilson et al hypothesize that environmental factors such as different viral inoculum, route of viral infection, and nutritional status can contribute to individual susceptibility of disease presentation.^[10]^ They speculated that amino acid substitutions resulted from *RANBP2* mutation might cause alteration of protein function and variations in the temperature sensitivity. Therefore, the severity and duration of fever may potentially contribute to incomplete penetration.^[8,10]^ As described earlier, clinical manifestation showed a wide spectrum in this family. Two male siblings were severely affected and deceased, and 1 male sibling had recurrent episodes, while the mother and female sibling did not have clinically apparent neurologic symptoms. It may be inferred that the female sibling might have been affected subclinically since focal brain lesions in both external capsules were incidentally detected during the head trauma evaluation.

Neuroimaging findings are more distinctive than clinical manifestations. Typical appearance of the ANE is symmetrical, multifocal T2 hyperintense lesions in the cerebral white matter, cerebellar medulla, upper brainstem tegmentum, and, especially, bilateral thalamus.^[11]^ In addition, most of the ANE lesions have characteristic appearances in DWIs called “tricolor pattern” or “target appearance,” which are apparent on ADC.^[6]^ The central portion of the lesions shows high ADC values due to edematous change; whereas, the peripheral portion shows low ADC values owing to vascular congestions and acute swelling of the oligodendrocytes. The outermost portion shows high ADC values as a result of extravasation. These findings are known to be frequent in the gray matter and thalamus.^[6]^ As forementioned, MR images of Case 1 and Case 2 showed symmetric involvement of abnormal signal intensity in the characteristic locations of the brain. Case 1 should have been diagnosed sooner if radiologists had known the typical tricolor pattern. MR images in Case 3 were consistent with brain death and did not show any diffusion restriction. It may be inferred that delayed imaging timing is responsible for such negative diffusion restriction.

Studies evaluating treatment option of familial ANE is limited since the familial ANE is a rare disorder. Early intensive care, supportive care such as seizure treatment and increased intracranial pressure control, antiviral therapy, intravenous corticosteroids, IVIG, and immunomodulatory agents are available treatment options.^[6,12]^ Administration of steroid at the early stage of the disease has been reported to improve clinical manifestation by reducing cytokine storm and metabolic dysfunction with relieving inflammation. However, the efficacy of IVIG, plasma exchange, or antiviral agents has not been proved in the literature.^[8]^

Prognosis of ANE ranges from complete recovery to death, as shown in our cases. Approximately 30% of mortality has been reported, and less than 10% of patients recovered completely with frequent neurological sequelae. Unilateral thalamic involvement and reversal of imaging findings are associated with a better outcome.^[6,8]^ On the contrary, early presentation <1-year-old, delirious behaviors associated with brain stem lesions, hemorrhagic lesions and cavitation on imaging studies, and elevated serum aminotransferase and CSF protein are associated with poor prognosis.^[6,8]^ Early treatment with high dose steroid is associated with improved outcomes, especially in patients without brain stem lesions.^[8,13]^

In conclusion, the authors report various radiological manifestations of familial ANE with *RANBP2* mutation in members of a Far-East Asian family. Since prompt diagnosis is paramount to patient management, familial ANE should be included in differential diagnoses when patients with recurrent neurological attacks after febrile respiratory illness show bilateral symmetrical brain lesions with characteristic diffusion restriction patterns in radiological examinations.

## Author contributions

**Conceptualization:** Jae-Yeon Hwang.

**Investigation:** Yu-Jung Park, Jae-Yeon Hwang, Yun-Jin Lee, Ara Ko.

**Supervision:** Jae-Yeon Hwang, Yong-Woo Kim.

**Writing – original draft:** Yu-Jung Park.

**Writing – review & editing:** Jae-Yeon Hwang, Yong-Woo Kim, Yun-Jin Lee, Ara Ko.
